# Nutraceuticals in prostate cancer therapeutic strategies and their neo-adjuvant use in diverse populations

**DOI:** 10.1038/s41698-018-0058-x

**Published:** 2018-07-25

**Authors:** Dominique Reed, Komal Raina, Rajesh Agarwal

**Affiliations:** 1 Department of Pharmaceutical Sciences, Skaggs School of Pharmacy and Pharmaceutical Sciences, Aurora, CO USA; 2 University of Colorado Cancer Center, University of Colorado Anschutz Medical Campus, Aurora, CO USA

## Abstract

Prostate cancer (PCa) is the most frequently diagnosed malignancy and second leading cause of cancer mortality in American males. Notably, men of African descent in the United States and Caribbean have the highest PCa mortality rates compared to men with European ancestry. Although current therapeutics are quite potent and effective, disease resistance, progression to metastasis, therapy-associated toxicities and efficacy-related issues in diverse populations develop over time. Thus, non-toxic and efficacious therapeutic strategies are needed to address these major obstacles for the clinical treatment and management of PCa. In this regard, preclinical and population-based efficacy studies have shown the potential of natural non-toxic nutraceuticals as potent anti-PCa agents. Accordingly, the implementation of nutraceutical intervention and genetic testing in diverse populations might aid in the development and design of precision medicine strategies to reduce the burden of chemotherapy-associated toxicities, suppress disease resistance, and treat both localized and advanced PCa. Consequently, additional large-scale and inclusive clinical studies are required to fully assess efficacy and therapeutic limitations of these agents in PCa. This review discusses the most current clinical research on selected nutraceutical agents and their efficacy in the context of clinico-pathological outcomes and disease susceptibility in diverse PCa clinical and epidemiological studies.

## Introduction

Despite recent advances in the treatment strategies used for the clinical management of metastatic prostate cancer (PCa), disease survival remains lower than 30%; notably, PCa is the second leading cause of cancer mortality in American men.^1^ Primary interventions for PCa include surgery, adjuvant chemotherapy, hormonal therapy (i.e., androgen deprivation), and/or radiation for advanced disease. Although these therapeutic options are quite potent and effective, disease resistance, progression to metastasis stage, therapy-associated toxicities and efficacy-related issues in diverse populations have developed over time. Thus, efficacious and non-toxic therapeutic strategies are needed to address these major obstacles for the clinical treatment and management of PCa. Past and ongoing chemoprevention/intervention research has recognized the use of nutraceutical agents as a feasible and non-toxic option, which could protect against tumorigenesis as well as enhance the therapeutic response of pre-existing anti-cancer treatments.^2^

**Fig. 1:**
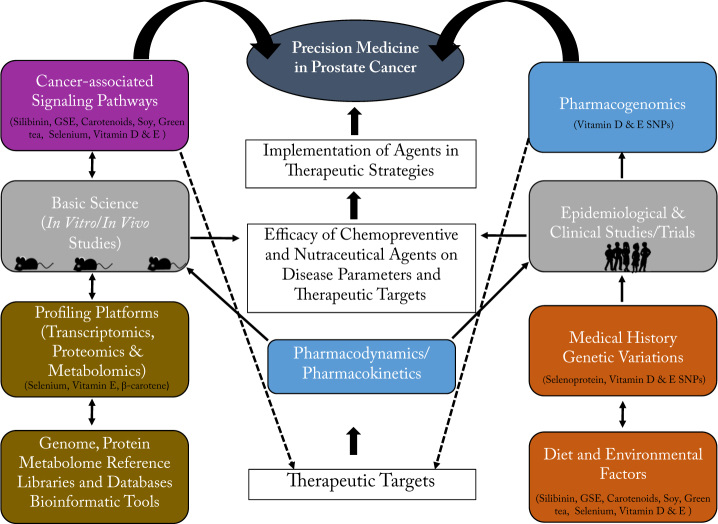
Nutraceutical efficacy in precision medicine. The schematic above depicts a workflow of experimental designs and assessment parameters required to establish the efficacy of nutraceutical agents. Therapeutic potential of agents must undergo vigorous pharmacodynamics and pharmacokinetics (blue) evaluation of their antioxidant and anti-cancer properties (e.g., anti-proliferation, anti-growth, anti-motility, anti-invasion) in cell models (gray). Targets that regulate altered tumor phenotypes are assessed via cell-focus assays (qRT-PCR, western blot, and immunofluorescence) and high-throughput platforms. Network analyses of targets are performed using omic profiling databases and libraries (brown) to determine gene ontology and identify therapeutic targets in cancer-associated canonical/non-canonical pathways (purple). Therapeutic targets and nutraceutical agents are evaluated in preclinical models and undergo the previous workflow. Next, nutraceutical agents are assessed in epidemiological studies and clinical trials (gray) which can evaluate hereditary, genetic, and environmental factors that range in degree from Phase I (≤30 patients, Pharmacodynamics/Pharmacokinetics parameters), Phase II (2–3 treatment groups including standard treatment + new agent, different doses, safety and toxicity assessments, and Pharmacogenomics in humans or animals), Phase III (comparison between new agent and standard treatments, Pharmacogenomics (blue)) (gray), Phase IV (Pharmacogenetic testing and side effects in different populations) to marketing and therapeutic application. After clinical trials have assessed the efficacy of nutraceuticals, these agents can be implemented in current precision medicine clinical therapeutic strategies for patients. The images of the mice and the group of people shown here are created by the authors.

Precision medicine using a nutraceutical approach is one of the key paradigms for the conceptualization and development of non-toxic therapeutic strategies that act synergistically with existing clinical anti-cancer agents. Precision medicine strategies navigate the intricate interplay between genetic, racial, and socio-economic factors, and involve interventions that compensate for individual variations in pharmacogenomic responses to cancer therapies and major PCa risk factors (i.e., age, family history, race, and genetic susceptibility) shown in Fig. 1. Genetic dispositions are critical for identifying individuals at higher risk for disease development or advanced disease and predicting therapeutic response to certain anti-cancer agents. Genetic variations have also shown protective and negative effects on PCa susceptibility. Thus, the efficacy of nutraceutical interventions is heavily influenced by ethnicity and genetic variations of patients. Overall, the relationship between nutraceutical agents, genetic variations, and other chemotherapeutic agents in different populations are not well understood in chemoprevention. Precision medicine strategies could provide a platform to address efficacy issues in PCa chemoprevention/intervention utilizing both chemotherapeutic and nutraceutical agents; however, additional clinical population studies are needed to confirm their efficacy against genetic, dietary, and environmental factors associated with PCa.

Precision medicine strategies utilize genomic analysis of PCa patients to identify and elucidate the pharmacogenomic landscape of patient susceptibility for the implementation of adjunct agents that can enhance the anti-tumor activity of pre-existing chemopreventive agents against advanced and/or resistant disease. This approach can aid in designing more specific treatment regimens for high-risk PCa patients. This review will evaluate the most current clinical research (summarized in Tables 1 and 2) on selected nutraceutical agents (silibinin, grapeseed extract, lycopene, soy isoflavanoids, green/black tea, vitamin E, vitamin D, zinc, and selenium) that may serve as adjunct agents in PCa. The efficacy of these nutraceutical agents will be discussed in the context of their effect on clinico-pathological outcomes (i.e., Gleason score, tumor grade, survival, biochemical recurrence), and disease susceptibility in diverse populations based on clinical and epidemiological data. We will also identify research gaps associated with diverse populations and selected nutraceuticals in PCa.

**Table 1:** Epidemiologic and clinical intervention studies with phytochemical nutraceuticals in prostate cancer

Natural product	Study type	Intervention	Population/location	Outcome	Reference
Silibinin	•Phase II •Non-randomized •Case-control cohort	•Daily oral administration of silybin-phytosome (13 g) in three doses daily prior to surgery •PCa patients aged ≥ 18 years with localized PCa scheduled for prostatectomy •Time: For 14–31 days	Univ. of Colorado Denver 12 PCa cases Ethnicity: N/a (Years: 2006–2010)	•High-dose oral silybin-phytosome achieves high blood concentrations (mean 19.7 µM) transiently, but low levels of silibinin are seen in prostate tissue (496.6 pmol/g) •No change in serum PSA, IGF-I, and IGFBP-3 levels •Low levels of silibinin in urine relative to plasma due to significant inter-patient variability	Flaig et al.^4^
Grape seed extract	•Case-control cohort	•Daily use of grape seed extract for 5–10 years •Men aged 50–76 years, (Surveillance, Epidemiology, and End Results (SEER) program cancer registry) filled base line questionnaire (and then followed for incident PCa) •Time: For 7 years and median follow-up time of 6.1 years	VITamins and Lifestyle (VITAL) study cohort Residents in the 13-county area of Western Washington State 1602 PCa cases;(35,239 total participants) Ethnicity: EA [1501 cases, 30,918 ctrls] AA [26 cases, 412 ctrls] Other [57 cases, 1876 ctrls]	•Examined the association between non-vitamin, non-mineral, “specialty” supplement use, and PCa risk •Grape seed extract decreased PCa risk by 41% (HR = 0.59, 95% CI = 0.40–0.86) •High 10-year average use of grape seed extract was associated with a 62% reduction in PCa risk (HR 0.38, 95% CI: 0.19–0.76) •Grape seed use was inversely associated with both low-grade (HR 0.58, 95% CI: 0.33–1.03) and high-grade PCa (HR 0.82, 95% CI: 0.34–2.00) compared to non-users	Brasky et al.^7^
Grape seed extract	•Phase II •Open-label, single-arm study	•Daily oral administration of grape seed extract (Leucoselect Phytosome, 300 mg) •Asymptomatic non-metastatic PCa patients with rising PSA •Adults ≥18 years of age •Histologically confirmed PCa. Evidence of rising PSA, baseline PSA must be ≥0.2 ng/mL at the time of screening •Time: For 1 year	Univ. of Colorado Denver/Cancer Center Recruitment in progress (2018)	•PSA levels will be measured every 6 weeks for 3 months, and then every 3 months up to 1 year	NCT03087903
Lycopene	•Phase I–II •Randomized, double-blind, placebo-controlled trial •Case cohort	•Daily administration of dietary selenium (55 µg), lycopene (35 mg), and green tea polyphenols (600 mg) •Patients diagnosed with multi-focal high-grade prostatic intraepithelial neoplasia (HGPIN) and/or atypical small acinar proliferation (ASAP) •Time: For 1 month (Phase I), 6 months (Phase II); mean follow-up of 37 months	Univ. of Turn, Italy 10 cases (Phase I), 60 cases (Phase II) Ethnicity: Italian (Years: 2009–2014)	•No significant variations in PSA (assessed by International Prostate Symptom Score questionnaire) •No significant change in mean PSA and DRE assessments after 6 months •Higher PCA diagnoses were in intervention group compared to placebo (*p* = 0.053) •Upregulated miRNAs (26b-5p, let-7i-5p, let-7d-5p, 16-5p, 199a-5p, 214-3p, 15a-5p, 29b-3p, 30e-5p, and 34a-5p) and downregulated miR-494, an oncosuppressor, in PCa relative to normal tissue	Gontero et al.^43^
Lycopene and *β*-cryptoxanthin	•Multi-disciplinary •Cross-sectional study •Case cohort	•Daily carotenoid intake (dietary and/or supplementation) assessed for 1 year •Prior to diagnosis of prostate adenocarcinoma in PCa patients (aged 40–79 years)	Data from North Carolina-Louisiana PCa project Ethnicity: AA [*n* = 1023] EA [*n* = 1079] (Years: 2004–2009)	•Total lycopene dietary and supplemental intake was inversely related to PCa aggressiveness in EAs (OR = 0.56, 95% CI: 0.34–0.90, highest vs. lowest tertile, *p*-trend = 0.03) •Dietary *β*–cryptoxanthin intake was inversely related to PCa aggressiveness in AAs (OR = 0.56, 95% CI: 0.36–0.87; *p*-trend = 0.01)	Antwi et al.^98^
β-cryptoxanthin, cis-lutein/zeaxanthin, and all-*trans*-lycopene	•Prospective •Randomized trial •Case cohort	•Weekly intervention of high intake of plant-based foods (whole grains, fruits, vegetables, and legumes (soybean products) and exercise) and low intake of meat and dairy products •Patients diagnosed with biochemically recurrent PCa •Time: For 6 months (3 months of active intervention followed by monthly boosters)	Midlands Region of South Carolina 39 cases Ethnicity: EA [*n* = 28 (72%)] AA [*n* = 11 (28%)]	•Plasma levels of β−cryptoxanthin (*p* = 0.01) and all-*trans*-lycopene (*p* = 0.004) were inversely related to PSA levels after dietary changes •Lower PSA levels at 3 and 6 months were associated with higher plasma levels of trans-β-carotene relative to baseline •High plasma levels of β−cryptoxanthin, cis-lutein/zeaxanthin, and all-*trans*-lycopene were associated with lower PSA levels after 6 months	Antwi et al.^14^
Lycopene-rich tomato products	•Randomized-controlled trial •Case cohort	•Daily administration of (a) tomato products containing lycopene (30 mg) per day; (b) Tomato products plus ([green tea (1 cup), black tea (1 cup), pomegranate juice (330 mL), grape juice (330 mL), soy-isoflavones (200 mg), 1-selenomethionin (200 µg), omega -3 fatty acids (3.13 g n-3 fatty acids)]; (c) control (habitual) diet •Prior to curative treatment of PCa patients with non-metastatic disease •Time: For 3 weeks	Oslo Univ. Hospital, Norway 86 cases Ethnicity: N/a (Years: 2007–2012)	•Tomato intervention alone decreased median serum PSA levels (−2.9%) significantly compared to controls (+6.5%) (*p* = 0.016) in the intermediate-risk group (post hoc analyses) •Highest plasma levels of lycopene alone decreased PSA (*p* = 0.009) •PSA levels were inversely related to lycopene plasma levels during and post intervention (*p* = 0.034, 0.048)	Paur et al.^11^
Lycopene/fish oil	•Three arm •Randomized, double-blinded placebo-controlled trial	•Daily administration of two lycopene (15 mg), three fish oil capsules [fish oil (1 gm), eicosapentaenoic (EPA) (1098 mg) and docosahexaenoic (DHA) (549 mg) fatty acids], and one multi-vitamin or placebo •Patients (young adult to older) with low burden PCa (Gleason Score sum ≤ 6, PSA ≤ 10 ng/mL, positive cancer biopsies ≤ 33%) •Time: For 90 days	Molecular Effects of Nutrition Supplements (MENS) Univ. of California San Francisco 84 patients Ethnicity: EA (78–83%) (Years: 2003–2008)	•High dietary intake of tomato was strongly associated with changes in Selenoamino Acid Metabolism (*p* = 0.0029) and androgen/estrogen metabolism for both high intake of tomato and fish in morphologically normal prostate tissue •Lycopene and fish oil supplementation were linked to alterations in nuclear factor (erythroid derived-2) factor 2 or Nrf2-mediated oxidative stress response signaling	Magbanua et al.^29^
Lycopene-rich tomato extract	•Phase II •Randomized, double-blind, placebo-controlled trial	•Daily administration of 2 capsules of Lyc-O-Mato [tomato oleoresin, 15 mg of lycopene, phytoene (1.4 mg), phytofluene (1.1 mg), β-carotene (0.7 mg), and α-tocopherol (4 mg)] or placebo (medium-chain triglycerides and red food coloring) •Patients (aged 35–75 years) diagnosed with high-grade prostatic intraepithelial neoplasia (HGPIN) (no cancer, atypical small acinar proliferation (ASAP), or antioxidant supplement use) •Time: For 6 months	Northwestern Memorial Hospital and the Jesse Brown Veterans Administration Medical Center, Chicago 58 patients Ethnicity: EA [*n* = 42 (19 treatment, 23 placebo)] AA [*n* = 15 (6 treatment, 9 placebo)], Other [*n* = 1(1 treatment)] (Years: 2006)	•High expression of MCM-2 in basal epithelial cells, p27 in luminal epithelial cells in benign prostate tissue •HGPIN prevalence post treatment was slightly decreased, but number of patients with extensive focal atrophy (≥5 biopsy cores) in the lycopene group •No differences in PSA, IGF-1, and IGFBP3 serum levels between intervention and placebo groups •No change in prevalence of PIA and inflammation in the lycopene group (*p* = 0.05)	Gann et al.^28^
β-carotene/other agents	•Large population based •Randomized, double-blind, placebo-controlled trial	•Daily administration of a capsule containing either a placebo or a combination of vitamin C (120 mg), α-tocopherol (30 mg), β-carotene (6 mg), selenium (100 µg), and zinc (20 mg) •Middle-aged patients without severe health problems •Time: For 8 years	SU. VI. MAX trial France and Canada 5141 patients Ethnicity: Caucasian (Years: 1994–2002)	•Reduced rate of PCa by 48% (HR = 0.52; 95% CI = 0.29–0.92; *p* = 0.009) among men with normal baseline PSA (<3 µg/L) •Non-significant increase in PCa incidence (HR = 1.54, 95% CI = 0.87–2.72) in men with elevated PSA levels (≥3 µg/L) •No effect on serum PSA and IGF levels	Meyer et al.^18^
β-carotene	•Randomized, double-blind, placebo-controlled trial	•Daily administration of α-tocopherol (50 mg), β-carotene (20 mg), both α-tocopherol and β-carotene, or placebo •Patients aged 50–69 years with a smoking history •Time: For 5 years, median follow of 6.1 years, follow-up of 18 years	Alpha-Tocopherol, β-Carotene Cancer Prevention (ATBC) Study 25,563 patients in Southwestern Finland Ethnicity: Finnish (Years: 1985–1993; National Registries follow-up till 2011)	•β-carotene intake increased post trial PCa mortality (RR = 1.20; 95% CI = 1.01–1.42) relative to non-recipients •No significant effect of β-carotene intake on PCa incidence	Virtamo et al.^26^
Soy	•Randomized double-blind, placebo-controlled trial	•Daily administration of soy isoflavone capsules [total isoflavones (80 mg/day), aglucon units (51 mg/day)] •Patients diagnosed with localized PCa •Time: For 6 weeks prior to prostatectomy	Univ. of Kansas Hospital and Kansas City Veteran Affairs, Medical Center, 86 cases Ethnicity: EA *n* = 69 [38 (90%) treatment, 31 (70%) ctrls] AA *n* = 12 [3 (7%) treatment, 9 (20%) ctrls] American Indian or Alaska Native *n* = 3 [1(2%) treatment, 2 (5%) ctrls] (Years: 2006–2009)	•Downregulated cell cycle and apoptotic-associated genes in prostate tumor tissue •No effect on serum levels of total testosterone, free testosterone, total estrogen, estradiol, PSA, and total cholesterol	Hamilton et al.^110^
Soy	•Phase II •Randomized trial •Case cohort	•Daily consumption of soy bread [2 slices (34 mg soy isoflavones/slice)] •Patients diagnosed with asymptomatic biochemically recurrent PCa •Time: For 8 weeks	Ohio State Univ. Medical Center, Columbus, Ohio 32 cases Ethnicity: N/A	•Decreased plasma levels of pro-inflammatory cytokines, Th1, T regulatory (CD4^+^CD25^+^FoxP3^+^), and monocytic (CD33^+^HLADR^neg^CD14^+^) myeloid-derived suppressor cells (MDSC) •Increased CD56^+^ Natural Killer (NK) cells	Lesinski et al.^32^
Soy	•Phase II •Randomized trial •Cross-over design	•Daily consumption of soy bread [2 slices (34 mg soy isoflavones/slice)] or soy-almond bread [2 slices (60 mg aglycone equivalents/day] •Patients diagnosed with asymptomatic biochemically recurrent PCa •Time: For 20 weeks (8 weeks intervention with one type of bread with 2 weeks of legume-free wash-out period)	Ohio State Univ. Medical Center, Columbus, Ohio 32 cases Ethnicity: N/A	•Increased blood levels of IGFBP-3 •Increased (3-fold) serum PSA doubling time •Decrease in LDL cholesterol and triglyceride levels in PCa patients with hypercholesterolemia	Anh Jarvis et al.^31^
Soy	•Randomized double-blind, placebo-controlled •Open-label trial	•Daily administration of GCP [genistein (450 mg), daidzein (300 mg), and other isoflavones)] •Patients with histologically confirmed PCa and two consecutive increases in PSA readings •Time: For 12 months	Univ. of California, Davis Medical Center 66 cases Ethnicity: GCP group EAs [*n* = 20] AAs [*n* = 3]	•High serum concentrations of genistein and daidzein but no significant difference in serum PSA levels after 6 and 12 months	De Vere et al.^111^
Soy	•Phase II •Randomized, double-blind placebo-controlled trial	•Daily administration of synthetic genistein (30 mg) •Patients diagnosed with localized PCa (aged ≥18 years) •Time: For 3–6 weeks prior to prostatectomy	Oslo Univ. Hospital, Norway 54 cases Ethnicity: Norwegian	•Decreased serum PSA levels (7.8%) relative to placebo treatment (*p* = 0.051) •Decreased blood levels of total cholesterol •No effect on thyroid or sex hormone blood levels	Lazarevic et al.^112^
Soy	•Pilot study •Case cohort	•Daily administration of soy isoflavone (200 mg) or placebo beginning with first day of radiation therapy (1.8–2.5 Gy) fractions for a total of 73.8–77.5 Gy •Patients aged ≥18years with localized PCa •Time: For 6 months	Wayne State Univ., Detroit, Michigan 42 cases Ethnicity: N/A	•Decreased serum PSA levels after pretreatment and radiation therapy in intervention group •Reduced adverse urinary, bowel, and rectal cramping symptoms induced by radiation in patients •After 3 months, less urinary incontinence, urgency, and better erectile function in patients	Ahmad et al.^113^
Soy	•Pilot •Randomized, double-blind, placebo-controlled trial •Case cohort	•Daily administration of soy protein [total isoflavones (160 mg)] or placebo •PCa patients undergoing medical or surgical ADT •Time: For 12 weeks	USA 33 cases Ethnicity: EA [*n* = 26 (15 treatment, 11 placebo)], AA [*n* = 7 (2 treatment, 5 placebo)]	•No significant change in serum PSA levels and lipid profiles •No effect on metabolic or inflammatory parameters	Napora et al.^114^
Soy	•Phase II trial •Case cohort	•Daily consumption of soy beverage (500 mL) •PCa patients with rising PSA after curative radiation •Time: For 6 months	USA, North America 34 cases Ethnicity: N/A	•Decreased serum PSA levels in four patients (13.8%) •Increased prolongation of serum PSA doubling time in eight patients (27.6%)	Kwan et al.^115^
Soy	•Randomized placebo-controlled double-blind trial	•Daily consumption of a supplement containing isoflavones (40 mg) and curcumin (100 mg) or placebo •Men with prostate biopsies (but no PCa) •Time: For 6 months	Teikyo Univ. School of Medicine, Tokyo, Japan 85 patients Ethnicity: Japanese	•Decreased serum PSA levels in patients with high PSA (PSA ≥10 ng/mL) (*p* = 0.01)	Ide et al.^116^
Soy protein isolate	•Randomized placebo-controlled trial	•Daily two doses of 1 of 3 soy protein isolates (40 g of protein): (1) soy protein (SPI+, 107 mg of isoflavones), (2) alcohol-washed soy protein (SPI–, <6 mg of isoflavones), or (3) milk protein (MPI) •High-risk PCa patients who undergone a transrectal ultrasound and biopsy, undergo active surveillance or diagnosed with high-grade prostatic intraepithelial neoplasia (HGPIN) or atypical small acinar proliferation (ASAP) •Time: For 6 months	Minneapolis Veteran’s Administration Medical Center, Univ. of Minnesota 58 patients Ethnicity: N/A	•Decreased Bax expression in prostate tissue from SPI group relative to MPI group (*p* = 0.03), but no changes in EFGr, Bcl-2, Bax:Bcl-2 or Bax:PCNA ratios among treatment groups •Increased prostate volume in SPI-group compared to MPI group •Supplementation had no effects on total PSA, free PSA, PSA percent, or PSA density •PCa incidence was six times higher in the MPI group than both soy treatment groups combined	Hamilton-Reeves et al.^36^
Soy	•Pilot •Randomized double-blind placebo-controlled trial	•Daily administration of three soy isoflavone tablet (Novasoy (genistein to daidzein (1.0:1.3)); 27.2 mg isoflavone aglycones) or a placebo •Patients newly diagnosed with PCa who will undergo prostatectomy and received no therapy •Time: 2 weeks prior to prostatectomy and follow-up after 3 years	Stanford Univ. School of Medicine, California 25 patients Ethnicity: N/A	•Decreased COX-2, prostaglandin (PG) receptors (EP4 or FP) expression in human PCa cell lines (LNCaP, PC-3) and primary prostate epithelial cells •Increased 15-hydroxyprostaglandin dehydrogenase (15-PGDH) mRNA levels and reduced PGE2 secretion in primary prostate cells •Suppressed basal and PG-stimulated growth and growth factor stimulation of COX-2 promoter in LNCaP cells •Decreased COX-2 and increased p21 mRNA levels in soy isoflavone-treated group •COX-2 expression negatively and p21 levels positively correlated with serum soy isoflavone levels in soy isoflavone-treated patients •Decreased protein levels of COX-2 in soy isoflavone group •Higher rate of disease recurrence among placebo patients after 3 years compared to soy isoflavone group	Swami et al.^35^
Tea polyphenol (Green tea)	•Phase 1–II •Randomized double-blind placebo-controlled trial •Case cohort	•Daily administration of dietary supplement [selenium (55 mg), lycopene (35 mg), and green tea catechins (600 mg)] or placebo •Patients diagnosed with multi-focal high-grade prostatic intraepithelial neoplasia (mHGPIN) and/or atypical small acinar proliferation (ASAP) •Time: For 1 month (Phase I), 6 months (Phase II), mean follow-up of 37 months	Univ. of Turin, Italy 10 cases (Phase I), 60 cases (Phase II) Ethnicity: Italian (Years: 2009–2014)	•No significant change in mean serum PSA levels •Higher PCa diagnoses were in intervention group at re-biopsy compared to placebo (*p* = 0.053) •Upregulated miRNAs (26b-5p, let-7i-5p, let-7d-5p, 16-5p, 199a-5p, 214-3p, 15a-5p, 29b-3p, 30e-5p, and 34a-5p) and downregulated miR-494, an oncosuppressor, in PCa relative to normal tissue	Gontero et al.^43^
Tea polyphenol (Green tea)	•Randomized, double-blind, placebo-controlled trial •Case cohort	•Daily administration of Polyphenon E (800 mg) or placebo •PCa patients (PSA <50 ng/mL) who received no therapy and scheduled for radical prostatectomy •Time: For 3–6 weeks prior to surgery	Arizona Cancer Center, Tucson, and NCI 48 cases Ethnicity: White [*n* = 45] Native American [*n* = 1] Other [*n* = 2] (Years: 2007–2010)	•No significant effect on serum PSA, and insulin-like growth factor levels •No change in proliferative and angiogenesis biomarkers expression in prostate tissue	Nguyen et al.^41^
Tea polyphenol (Green/Black tea)	•Phase II •Open-label •Prospective randomized trial •Case cohort	•Daily intake of brewed green tea [6 cups, EGCG (562 mg)] and/or black tea [6 cups, EGCG (28 mg), theaflavins (35 mg), Gallic acid (348 mg)] or control (water) •Patients with localized PCa and scheduled for radical prostatectomy •Time: For 3–8 weeks prior to surgery	Veterans Administration Greater Los Angeles (LA) Health System; Univ. of California (LA), UCLA-Santa Monica Medical Center. 93 cases Ethnicity: White [*n* = 63] AA [*n* = 17] Asian [*n* = 4] Hispanic [*n* = 8] Other [*n* = 1]	•Reduced NFkB nuclear levels in radical prostatectomy tissue in green tea cohorts (*p* = 0.013) but not black tea (*p* = 0.931) •Green tea decreased serum PSA levels and oxidative DNA damage marker in urine	Henning et al.^40^
Tea polyphenols (EGCG)	•Phase II •Open-label, single-arm two-stage trial	•Daily administration of Polyphenon E [(EGCG (800 mg), with lesser amounts of (−)- epicatechin, (−)-epigallocatechin, and (−)-epicatechin-3-gallate)] •Patients aged 18–75 years diagnosed with positive prostate biopsies and scheduled for radical prostatectomy •Time: For 6 weeks prior to surgery	USA 26 patients Ethnicity: AA [*n* = 16 (62%)] EA [*n* = 10 (38%)]	•Polyphenon E decreased serum levels of PSA, HGF, VEGF, IGF-I, IGFBP-3, and the IGF-I/IGFBP-3 ratio in PCa patients •Race had no significant effect in relation to intervention on serum biomarkers	McLarty et al.^38^
Tea polyphenol (Green tea)	•Double-blind •Randomized placebo-controlled trial •Case cohort	•Daily administration of oral capsule containing [pomegranate (100 mg), green tea (100 mg), broccoli (100 mg), and turmeric (100 mg)] •Patients diagnosed with localized PCa disease and undergoing active surveillance or watchful waiting •Time: For 6 months	UK-NCRN Pomi-T study 199 cases Ethnicity: N/A (All across UK) (Years: 2011–2012)	•Supplement intervention decreased median rise in serum PSA levels in PCa patients (*p* = 0.0008) •Reduced serum PSA levels in men receiving either active surveillance or watchful waiting (*p* = 0.001) •No differences in Gleason grade, cholesterol, blood pressure, blood sugar, C-reactive protein in groups	Thomas et al.^39^
Tea polyphenol (Green and Black tea)	•Case cohort	•Daily administration of 1.42 L Green tea (EGCG), Black tea (theaflavin), or a caffeine-soda control (SC) •Patients with localized PCa •Time: For 5 days prior to prostatectomy	VA Greater Los Angeles Healthcare System (VAGLAHS) Univ. of Los Angels 20 cases Ethnicity: N/A	•Relative absorption of theaflavin 70% greater tbat EGCG *in vivo*, but higher levels were observed of EGCG and ECG-conjugated forms in the small intestine, liver, and prostate •In the colon, polyphenols and theaflavins were present as theitr free forms •Tea polyphenols were not detected in patient serum •Serum from patients treated with Green or Back tea reduced LNCaP proliferation relative to control serum	Henning et al.^42^

*EA* European-American, *AA* African-American, *PCA* prostate cancer, *PSA* prostate-specific antigen, *BPH* benign prostatic hyperplasia, *HR* hazard ratio, *OR* odds ratio, *RR* relative risk, *DRE* digital rectal exam, *ADT* androgen deprivation therapy, *SNP* single-nucleotide polymorphism, *IGF-I* insulin-like growth factor-I, *IGFBP-3* IGF-binding protein-3, *miRNA* microRNA, *Ctrls* controls

**Table 2:** Epidemiologic and clinical intervention studies with vitamin and trace element/mineral-associated nutraceuticals in prostate cancer

Natural product	Study type	Intervention	Population/location	Outcome	Reference
Vitamin D	•Case-control cohort	•Daily oral administration vitamin D3 (4000 IU) •PCa patients scheduled for prostatectomy •Time: For 2 months prior to surgery	USA 27 cases Ethnicity:AA (*n* = 10) EA (*n* = 17)	•Reduced immune and inflammation signaling in PCA transcriptome •Increased expression of immune and inflammation-associated genes in AAs relative to EAs	Hardiman et al.^54^
Vitamin D	•Phase I •Open-label multi-center, non-randomized dose-escalation study	•Oral administration of inecalcitol (40–8000 µg) daily, or twice a day in combination with a 1-h intravenous infusion of docetaxel (75 mg/m^2^, every 3 weeks) and oral prednisone (5 mg twice a day) •Naive metastatic castrate-resistant PCa patients received up to six 21-day treatment cycles •Time: For 6 months	France 54 cases Ethnicity: French	•Reduced serum PSA levels (at 4000 µg) by ≥30 and ≥50% within the first 3 months •Increased median time of PSA progression to 169 days •Well tolerated at 4000 µg	Medioni et al.^52^
Vitamin D	•Phase IIa •Randomized placebo-controlled trial	•Daily oral administration of cholecalciferol (vitamin D3 200,000 IU) as one dose at study entry plus genistein [(G-2535), 600 mg daily], or placebo cholecalciferol day 1 and placebo genistein daily •Patients with early stage PCa and scheduled for radical prostatectomy •Time: For 21–28 days prior to surgery	Univ. of Wisconsin chemoprevention consortium 15 cases Ethnicity: N/A	•Non-significant increase in calcitriol serum concentrations compared to placebo (0.104 ng/mL ± 0.2 vs. 0.0013 ng/mL ± 0.08; *p* = 0.08); but no increase in prostate tissue •Elevated AR expression (*p* = 0.04) and apoptosis (*p* = 0.1) in prostate tumor tissues compared to placebo	Jarrad et al.^51^
Vitamin D	•Phase III •Multi-center •Randomized open-label study •Case cohort	•Oral administration of ASCENT [high-dose calcitriol (45 µg), docetaxel (36 mg/m^2^), and dexamethasone (24 mg)] for 3 out of every 4 weeks or control [prednisone (5 mg) twice daily with docetaxel (75 mg/m^2^), and dexamethasone (24 mg) every 3 weeks) •Patients diagnosed with metastatic castration-resistant PCa and disease progression •Time: follow-up of 48 weeks	ASCENT Study 198 hospitals [USA, Canada, Germany, Hungary, Czech Republic, Romania, Slovakia, and Serbia] 953 cases North American (722 cases), Europe (231 cases) (Years: 2006–2007)	•More deaths in ASCENT arm and trial was halted •Decreased median overall survival to 17.8 months (95% CI = 16.0–19.5) compared to 20.2 months (95% CI = 18.8–23.0) in the control arm (log-rank *p* = 0 .002) •Survival remained inferior after adjusting for baseline variables (HR = 1.33, *p* = 0.019) •No difference in serum PSA levels •Docetaxel toxicity occurred by 2-fold in ASCENT (31%) compared to control arm (15%) •Common adverse events were GI effects (75% of patients), blood and lymphatic disorders (48%)	Scher et al.^117^
Vitamin D	•Case-cohort design nested within SELECT	•Daily administration of selenium (200 μg of L-selenomethionine) + vitamin E (400 IU of *all rac*-α -tocopheryl acetate), vitamin E and placebo, selenium and placebo, and placebo •Patients (aged ≥50 years (AA) or ≥55 years) with no PCa history, PSA ≤ 4 ng/mL and non-abnormal DRE •Time: For 7–12 years, median overall follow-up of 5.46 years	Data from Selenium and Vitamin E Cancer Prevention Trial (SELECT) 427 sites (United States, Canada, and Puerto Rico) 1731 cases, 3203 cohort Ethnicity: EA [*n* = 1394 (80.5%) cases, *n* = 2213 (69.1%) cohort] AA [*n* = 250 (14.4%) cases, *n* = 802 (25%) cohort] Other [*n* = 87 (5%) cases, *n* = 188 (5.9%) cohort]	•Both low and high vitamin D concentrations were associated with increased risk of PCa, and more strongly for high-grade disease •Optimal range of circulating vitamin D for PCa prevention may be narrow; significantly reduced risks among men with moderate vitamin D concentrations (approximately 45–70 nmol/L) •Higher vitamin D levels associated with reduced risk of advanced PCa in AAs	Kristal et al.^118^
Vitamin D	•Phase II •Randomized, double-blind, placebo-controlled cancer	•Daily oral administration of vitamin D3 (cholecalciferol) at doses 400, 10,000, or 40,000 IU •Patients (30–85 years) with a Gleason score 6 or 7 •Time: For 3–8 weeks prior to prostatectomy	University Health Network 45 patients University of Toronto Toronto, Canada Ethnicity: N/A (Years: 2008–2012)	•Prostatic (1,25(OH)2D) concentrations showed that VDR was significantly lower in prostate tissues with the highest concentration of 1,25(OH)2D •IL-6 expression was the highest in the prostate stroma, while PTGS2 (COX2) levels were lowest in the prostate cancer tissues from men in the highest tertile of prostatic 1,25(OH)2D •TNF-α, IL-6, and IL-8 were suppressed by 1,25 (OH)2D in the primary epithelial cells, whereas TNF-α and PTGS2 were suppressed by 1,25(OH) 2D in the stromal cell	Giangreco et al.^63^
Vitamin E	•Randomized, double-blind, placebo-controlled cancer prevention trial	•Daily administration of α-tocopherol (*all-rac-α-*tocopheryl acetate 50 mg) or β-carotene (20 mg) or both α-tocopherol and β-carotene, or placebo •Patients aged 50–69 years with a smoking history •Time: For 5 years, median follow of 6.1 years, follow-up of 18 years	Alpha-Tocopherol, β-Carotene Cancer Prevention (ATBC) Study 25,563 patients in Southwestern Finland Ethnicity: Finnish (Years: 1985–1993; National Registries follow-up till 2011)	•α-tocopherol reduced post-trial PCa mortality (RR = 0.84; 95% CI = 0.70–0.99) relative to non-recipients •Daily α-tocopherol (50 mg) for a median of 6.1 years decreased the risk of PCa •No significant late effects (follow-up of 18 years) of α-tocopherol intake on PCa incidence	Virtamo et al.^26^
Vitamin E	•Randomized, double-blind, placebo-controlled trial •Case-control cohort	•Daily oral administration of selenomethionine (200 μg), vitamin E (*all-rac*-*α*-tocopheryl acetate, 400 IU), or both or placebo •Patients with no PCa history, PSA ≤ 4 ng/mL and non-abnormal DRE •Time: For 0–7.9 years, median follow-up of 5.5 years •The trial was stopped early due to lack of efficacy of either supplement	SELECT study 1746 PCa incident cases and sub-cohort of 3211 derived from SELECT trial 427 sites (United States, Canada, and Puerto Rico) Ethnicity: AA [*n* = 251(14.4%) cases, *n* = 735 (24.4%) ctrls] Hispanic [*n* = 58 (3.3%) cases, *n* = 139 (4.6%) ctrls] EA [*n* = 1406 (80.5%) cases, *n* = 2083 (69.2%) ctrls] Other [*n* = 31 (1.8%) cases, *n* = 51(1.7%) ctrls]	•High Pca incidence in men supplemented with high-dose α-tocopherol •Higher plasma α-tocopherol levels could interact with selenomethionine supplements to increase high-grade PCa risk •Higher PCa hazard risk (HR = 2.04, 95% CI = 1.29–3.22, *p*-trend = 0.005) was associated with patients who received selenomethionine supplement in combination with α-tocopherol in the fifth quintile relative to the first quintile •Plasma levels of α-tocopherol were positively associated with risk of high-grade PCa disease (HR = 1.59, 95% CI = 1.13–2.24, *p*-trend = 0.001, Gleason grade 7–10), in the fifth quintile •Plasma levels of α- and γ-tocopherol were not associated with PCa overall	Albanes et al.^67^
Vitamin E (APC-100)	•Phase I/ IIa •Open-label, non-randomized, dose-escalation study •Case cohort	•Oral administration of antioxidant moiety of vitamin E [APC-100, (900–2400 mg)] •Patients diagnosed with castrate-resistant PCa •Time: For 4–36 weeks	USA 20 cases Ethnicity: EA [*n* = 13 (65%)] AA [*n* = 5 (25%)] Asian [*n* = 2 (10%)]	•25% of patients receiving APC-100 treatment maintained stable disease •Median progression free survival) was 2.8 months •APC-100 undetectable in plasma at dose of 2100 mg	Kyriakopoulos et al.^64^
Vitamin E	•Double-blind, placebo-controlled cancer prevention trial	•Daily oral administration of α-tocopherol (*all-rac-α-*tocopheryl acetate 50 mg), β-carotene (20 mg), both or placebo •Patients aged 50–69 years with a smoking history •Time: For 5–8 years	Data from ATBC study 200 cases (100 aggressive) and 200 controls Southwestern Finland Ethnicity: Finnish (Years: 1985–1993)	•Serum metabolomic response to supplementation determined •Downregulated vitamin E (γ-tocopherol, β-tocopherol) and amino acid (N6-aceytllysine, β-alanine, ornithine, and glutarylcarnitine) metabolites •Upregulated vitamin E (α-tocopherol), co-factor (α-CEHC sulfate, α-CEHC glucuronide) and carbohydrate (fructose) metabolites	Mondul et al.^119^
Vitamin E	•Case cohort	•Daily intake/administration of vitamin E supplements (30, 100, 200, 400, 600, or 800 IU) assessed •Patients diagnosed with prostate adenocarcinoma and residing within the study catchment areas •Time: For 1 year prior to diagnosis	Data from North Carolina-Louisiana PCa project 2102 cases Ethnicity: AA [*n* = 1023] EA [*n* = 1079] (Years: 2004–2009)	•Dietary and supplemental α-tocopherol and PCa aggressiveness were inversely related in AAs (*p*-trend = 0.2, 0.15) •Dietary intake of α- (*p*-trend = 0.006) and δ-tocopherol (*p*-trend = 0.007) were related inversely to PCa aggressiveness among EAs	Antwi et al.^66^
Vitamin E	•Prospective •Randomized trial •Case cohort	•Weekly intervention of high intake of plant-based foods (whole grains, fruits, vegetables, and legumes (soybean products) and exercise) and low intake of meat and dairy products •Patients diagnosed with biochemically recurrent PCA •Time: For 6 months (3 months of active intervention followed by monthly boosters)	Midlands Region of South Carolina 39 cases Ethnicity: EA [*n* = 28 (72%)] AA [*n* = 11 (28%)]	•After adjusting for baseline PSA levels, plasma levels of α-tocopherol (*p* = 0.01) at 3 months were inversely related to serum PSA levels at 6 months •Lower serum PSA levels at 3 and 6 months were associated with percent increase in plasma levels of α-tocopherol relative to baseline	Antwi et al.^14^
Vitamin E	•Randomized trial	•Administration of vitamin E (400 IU) every other day, vitamin C (500 mg) daily, or their respective placebos •Physicians aged ≥ 50 years •Time: 10.3 years, post-trial follow-up of 2.8 years	Physicians Health Study II Total of 14,641 US Physicans enrolled 1373 PCa cases Ethnicity: N/A (Years: 1997–2007), follow-up till 2011	•Supplementation had no effect on PCA incidence	Wang et al.^70^
Vitamin E/lycopene	•Prospective •Randomized, double-blind, placebo-controlled trial	•Daily administration of α-tocopherol (50 mg), β-carotene (20 mg), both α-tocopherol and β-carotene, or placebo •Patients aged 50–69 years with a smoking history •Time: For 5 years, median follow of 6.1 years, follow-up of 18 years	Data from ATBC study 200 cases (100 aggressive) and 200 controls Southwestern Finland Ethnicity: Finnish (Years: 1985–1993)	•Energy and lipid-related serum metabolite levels were associated with low risk of aggressive PCa with the exception of Erucoyl-sphingomyelin and Trimethylamine N-oxide •Serum levels of other metabolite chemical classes were not associated to non-aggressive or overall PCa risk •Glycerophospholipid, long-chain fatty chain, and TCA metabolites were primarily modulated compared to other metabolites	Mondul et al.^73^
Vitamin E/selenium	•Randomized, placebo-controlled trial •Case-cohort	•Daily oral administration of selenomethionine (200 μg), vitamin E (*all-rac-α*-tocopheryl acetate, 400 IU), or both or placebo •Patients with no PCa history, PSA ≤ 4 ng/mL and non-abnormal DRE •Time: 7–12 years, median overall follow-up of 5.46 years	Data from SELECT study 1739 cases, 2922 ctrls Ethnicity: AA [13.7% cases, 24% ctrls] Hispanic [3.3% cases, 4.3% ctrls] EA [81.2% cases, 70% ctrls] Other [1.8% cases, 1.7% ctrls]	•Vitamin E supplementation increased the risk of PCa among men with low selenium status •Overall, low-grade, and high-grade PCa risk was higher among men with lower selenium status and receiving vitamin E supplements (*p* = 0.008) •Vitamin E supplementation (alone) had no effect in men with high selenium status (≥40th percentile of toenail selenium) (*p* = 0.02) •Selenium supplementation did not benefit men with low selenium status but increased the risk of high-grade PCa among men with high selenium status •Men should avoid selenium and vitamin E supplementation at doses that exceed recommended dietary intakes	Kristal et al.^68^
Vitamin E/selenium	•Case cohort study of SELECT trial participants	•Daily oral administration of selenomethionine (200 μg), vitamin E (*all-rac-α*-tocopheryl acetate, 400 IU), or both or placebo •Patients with no PCa history, PSA ≤ 4 ng/mL and non-abnormal DRE •Time: 7–12 years, median overall follow-up of 5.46 years	Data from SELECT study Sub-cohort: 1866 cases	•NKX3.1 rs11781886 genotypes did not significantly modify total low-grade or high-grade PCa risk •NKX3.1 rs11781886 genotypes CC (total, low grade) in the selenium arm and CT (total, high grade) in the vitamin E arm were associated with increased risk of PCa	Martinez et al.^72^
Vitamin E/selenium	•Case cohort study of SELECT trial participants	•Daily oral administration of selenomethionine (200 μg), vitamin E (*all-rac-α*-tocopheryl acetate, 400 IU), or both or placebo •Patients with no PCa history, PSA ≤ 4 ng/mL and non-abnormal DRE •Time: For 7–12 years, median overall follow-up of 5.46 years	Data from SELECT study Sub-cohort: 1434 cases	•The effect of selenium or vitamin E supplementation on high-grade PCa risk may vary by genotype •Inheritance of *TTPA* genetic variants (*rs12679996*, *rs4606052*) and Vitamin E supplementation were linked to increased PCa risk •SNPs in Vitamin E-associated genes, *SEC14L2*, *SOD1*, and *TTPA*, modified risk of developing high-grade PCa disease •Potential interactions between*SOD2*, *SOD3*, and *TXNRD2*, and selenium status and PCa risk	Chan et al.^94^
Selenium	•Phase III •Randomized doubled-blind placebo-controlled trial	•Daily oral administration selenium (200 µg /or 400 µg) or placebo •High-risk PCa patients (PSA > 4 ng/mL and/or abnormal DRE) with negative prostate biopsy •Time: Follow-up of 5 years	Negative Biopsy Trial (NBT)-USA and New Zealand 699 patients Ethnicity of 699 patients were N/A Ethnicity of initial participants White [*n* = 404 (86.7%)] AA [*n* = 16 (3.4%)] Asian [*n* = 5 (1.1%)] Hispanic [*n* = 38 (8.2%)] Native American [*n* = 3 (0.6%)]	•Intervention did not significantly modify PCa susceptibility •No changes in PSA velocity were seen in selenium treatment groups relative to placebo subjects •Mortalities occurred in the placebo (5), low (3), and high-dose selenium (2) treatment groups	Lu et al.^85^
Selenium	•Phase II •Randomized, double-blind, placebo-controlled trial •Case cohort	•Daily oral administration of 200/or 800 μg of selenium or placebo •Patients diagnosed with localized non-metastatic PCa (Gleason score < 8, PSA < 50 ng/mL, age < 85 years) •Time: For placebo (36.3 months, follow-up 38.4 months); selenium 200 μg (33.4 months, 33.3 months), 800 μg: (33.3 months, 33.8 months)	USA 140 cases Ethnicity: EA [*n* = 123 (88%)] Non-EA [*n* = 17 (12%)]	•No significant effects on PSA velocity and Gleason score •Increased PSA velocities in the highest quartile of patients receiving high-dose selenium (800 µg) relative to placebo (*p* = 0.018) •Total of four deaths in treatment groups	Lu et al.^85^
Selenium	•Double-blind, randomized, placebo-controlled trial	•Daily administration of selenium as selenomethionine 200 μg/day) or placebo •Men ≥40 years of age with a confirmed diagnosis of HGPIN lesions via biopsy with no evidence of PCa •Time: For 3 years •The primary endpoint was progression of HGPIN to PCa over a 3-year period	NCI Intergroup trial/Southwest Oncology Group (SWOG) 423 randomized men with HGPIN Ethnicity: N/A	•Selenium supplementation had no effect on PCa risk •No differences in Gleason scores between the two arms	Marshall et al.^120^
Selenium	•Phase I/II •Randomized double-blind placebo-controlled trial •Case cohort	•Daily administration of dietary supplement [selenium (55 mg), lycopene (35 mg), and green tea catechins (600 mg)] or placebo •Patients diagnosed with multi-focal high-grade prostatic intraepithelial neoplasia (mHGPIN) and/or atypical small acinar proliferation (ASAP) •Time: For 1 month (Phase I), 6 months (Phase II), mean follow-up of 37 months	Univ. of Turin, Italy 10 cases (Phase I), 60 cases (Phase II) Ethnicity: Italian (Years: 2009–2014)	•No significant change in mean serum PSA levels •Higher PCa diagnoses were in intervention group at re-biopsy compared to placebo (*p* = 0.053) •Upregulated miRNAs (26b-5p, let-7i-5p, let-7d-5p, 16-5p, 199a-5p, 214-3p, 15a-5p, 29b-3p, 30e-5p, and 34a-5p) and downregulated miR-494, an oncosuppressor, in PCa relative to normal tissue	Gontero et al.^43^
Selenium	•Randomized, double-blind, double-dummy trial •Multi-center	•Patient data from Profluss^®^ intake 1 tablet/day [(85% of fatty acids sterols, selenium(50 µg) and lycopene (5 mg)] and control •Patients aged 55–80 years diagnosed with lower urinary tract symptoms (negative DRE for PCa, PSA < 4 ng/mL) •Time: For 1 year, follow-up of 2 years	Post hoc analysis of Procomb trial 209 patients Ethnicity: Italian (Years: 2012–2014)	•No detrimental or protective role of supplementation in increasing PCa risk •No significant differences in the mean serum PSA levels or Gleason score •No effect on PCa risk (OR = 1.07; 95% CI = 0.64–1.79; *p* = 0.95), incidence (HR = 1.38; 95% CI = 0.32–5.90; *p* = 0.67)	Morgia et al.^27^
Selenium	•Randomized, placebo-controlled trial	•Daily oral administration of selenium (selenized yeast, 300 µg) or placebo •Patients undergoing diagnostic prostate biopsies and radical prostatectomy •Time: For 5 weeks	Netherlands 23 cases Ethnicity: Dutch	•Downregulated genes associated with cell migration, invasion, remodeling, and immunity •Exhibited an inhibitory effect against EMT via upregulation of epithelial markers (E-cadherin and EPCAM) and downregulation of mesenchymal markers (vimentin and fibronectin)	Kok et al.^90^
Selenium	•Randomized, double-blind, placebo-controlled trial •Case-control cohort •(sub-study of participants within SELECT)	•Daily oral administration of selenomethionine (200 μg) or vitamin E (*all-rac-α*-tocopheryl acetate, 400 IU), or both selenomethionine and vitamin E or placebo •Patients with no PCa history, PSA ≤ 4 ng/mL and non-abnormal DRE •Time: For 0–7.9 years, median follow-up of 5.5 years •The trial was stopped early due to lack of efficacy of either supplement	SELECT study data 1746 PCa incident cases and sub-cohort of 3211 derived from SELECT trial 427 sites (United States, Canada, and Puerto Rico) Ethnicity: AA [*n* = 251(14.4%) cases, *n* = 735 (24.4%) ctrls] Hispanic [*n* = 58 (3.3%) cases, *n* = 139 (4.6%) ctrls] EA [*n* = 1406 (80.5%) cases, *n* = 2083 (69.2%) ctrls] Other [*n* = 31 (1.8%) cases, *n* = 51(1.7%) ctrls]	•Increased PCa risk (HR = 2.04; 95% CI = 1.29–3.22) in patients receiving selenomethionine alone or in combination with α-tocopherol in the highest quintile relative to the first quintile (*p*-trend = 0.005) •Positively associated with plasma levels of α-tocopherol in patients receiving selenomethionine in the fifth quintile (HR = 2.12; 95% CI = 1.32–3.40; *p*-trend = 0.0002) •Non-significant elevation of PCa risk associated with selenomethionine in the third tertile of plasma α-tocopherol levels relative to placebo	Albanes et al.^67^
Selenium	•Randomized-controlled trial •Case cohort	•Daily administration of (a) tomato products containing lycopene 30 mg per day; (b) tomato products plus ([green tea (1 cup), black tea (1 cup), pomegranate juice (330 mL), grape juice (330 mL),soy- isoflavones (200 mg), 1-selenomethionin (200 µg), omega -3 fatty acids (3.13 g n-3 fatty acids)]; (c) control (habitual) diet •Prior to curative treatment of PCa patients with non-metastatic disease •Time: For 3 weeks	Oslo University Hospital, Norway 86 cases Ethnicity: N/A (Years: 2007–2012)	•Tomato products plus therapy slightly decreased (non-significant) serum PSA levels among intermediate-risk patients post surgery •Decreased serum PSA levels with highest increases in levels of lycopene, selenium and fatty acid C20:5 n-3 •Tomato products plus therapy significantly changed fatty acid profiles (*p* < 0.001) and doubled plasma selenium values	Paur et al.^11^
Selenium	•Pilot •Randomized, double-blind, placebo-controlled trial	•Daily administration of selenium-enriched yeast (SY) (247 μg) or placebo (non-enriched yeast) •Healthy subjects (aged 19–43 years) with no smoking history •Time: For 9 months, follow-up after 12 months	American Health Foundation, New York and Penn State College of Medicine, Pennsylvania 36 healthy patients Ethnicity: AA [*n* = 11 (31.6% ctrl, 29.4% treatment)] EA [*n* = 25 (68.4% ctrl, 70.6% treatment)]	•Upregulated (clusterin isoform 1 [CLU], transthyretin, α-1Bglycoprotein, transferrin, complement component 4B proprotein, isocitrate dehydrogenase, haptoglobin, keratin 1) and downregulated (α-1 antitrypsin [AAT], angiotensin precursor and albumin precursor) several proteins •CLU and AAT are associated with PCa development •Supplementation resulted in selenium plasma levels lower in AAs relative to EAs •Levels of AAT were higher in AA men compared to EA men •Supplementation reduced AAT levels; however, after intervention AAT levels recovered in AAs at 12 months, but remained low in EA men	Sinha et al.^91^
Selenium/lycopene	•Multi-center •Cohort	•Daily administration of Profluss [1:1 ratio of SeR 320 mg + lycopene (5 mg) + Selenium (50 μg) (group I), control (group Ic), SeR 320 mg + Lycopene (5 mg), Selenium(50 μg), and α-blockers treatment (group II), control (group IIc)] •Patients with LUTS, PSA > 4 ng/mL, Abnormal DRE/transrectal ultrasound, chronic inflammation-associated BPH, high-grade PIN and/or ASAP •Time: For 6 months (Group I) and 3 months (Group II)	Flogosis And Profluss in Prostatic and Genital Disease (FLOG) study 9 centers (Italy) 108 patients (Group I) 60 patients (Group II) Ethnicity: Italian (Years: 2009-2010)	•Decreased serum PSA levels in Group I, but no difference in Group II •Reduced extension and grading of flogosis in treated patients •Decreased of total interstitial mononuclear cells, mancrophages, B and T lymphocytes after 6 months in group I and 3 months in group II relative to controls	Morgia et al.^93^
Selenium/multi-vitamins	•Prospective cohort	•Daily intake of multi-vitamins or individual supplement (such as, selenium, β-carotene, and zinc) •PCa free at enrollment. Patients with AARP memberships •Time: For 1 year, follow-up to 5 years	National Institutes of Health (NIH)-AARP Diet and Health Study 10,241 cases (8765 localized and 1476 advanced disease) Ethnicity: EA (92%), AA (4%) Other (4%) (Years 1995–1996)	•Increased risk of advanced (RR = 1.32; 95% CI = 1.04–1.67) and fatal (RR = 1.98; 95% CI = 1.07–3.66) PCa with excessive use of multi-vitamins relative to non-users •Frequent use (≥7 per week) increased risk of PCa (*p*-trend = 0.003) and localized disease (*p*-trend = 0.004) •Strongly associated with PCa risk in patients with a family history of PCa	Lawson et al.^65^
Zinc	•Case-control surveillance cohort	•Daily use of multi-vitamin containing zinc, vitamin E, beta-carotene, folate, and selenium •Patients with a primary diagnosis of PCa and no other malignancy except non-melanoma skin cancer •Time: For 1–10 years or more	USA hospitals located in four centers (Baltimore, Boston, New York, and Philadelphia) 1706 cases, 2404 matched ctrls Ethnicity: EA [83.9% cases, 77% ctrls] AA [16.1% cases, 23% ctrls] (Years: 1976 onwards)	•10 years or more use of zinc in a multi-vitamin or supplement was linked to 2-fold (OR = 1.9, 95% CI = 1.0–3.6) increase in PCA risk •Lower risk was associated with the use of multi-vitamin that did not contain zinc	Zhang et al.^16^
Zinc	•Randomized, placebo-controlled trial	•Daily intake and supplemental use of vitamin C, vitamin D, zinc, calcium, carotenoids, lycopene, EPA* plus DHA* or multi-vitamin weekly •Placebo patients, free of BPH at baseline •Time: For 1 year	Prostate Cancer Prevention Trial (PCPT) subjects 4770 patients Ethnicity: EA [*n* = 4460 (93.5%)] AA [*n* = 153 (3.2%)] Hispanic [*n* = 98 (2.1%)] Other [*n* = 59 (1.2%)]	•BPH assessed by International Prostate Symptom Score questionnaire. Diet, alcohol, and supplement use assessed by food frequency questionnaire •Supplement intake did not affect BPH risk •A diet low in fat and red meat, and high in protein and vegetables, as well as regular alcohol consumption may reduce risk of BPH	Kristal et al.^17^
Zinc	•Prospective •Case-control cohort	•Daily use of vitamin E, selenium, and zinc supplements •Patients with no history of PCa •Time: Average follow-up 3.5 years	VITAL study cohort (USA) 832 cases, 34,412 ctrls Ethnicity: EA [781 (94.6%) cases, 31,642 (93.2%) ctrls] AA [17 (2.1%) cases, 421 (1.2%) ctrls]	•10 year average intake of supplemental zinc was not associated with a reduced PCa risk overall •Risk of advanced PCa (regionally invasive or distant metastatic) decreased with greater intake of supplemental zinc (adjusted HR = 0.34, 95% CI = 0.13–1.09 for 10-year average intake >15 mg/day vs. non-use, *p*-trend = 0.04)	Gonzalez et al.^77^
Zinc	•Multi-stage, stratified sampling design	•Daily zinc intake and cadmium exposure in relation to recommended daily allowance •Subjects aged 50 years or more (zinc intake and cadmium exposure fit within the tolerable range limit for adults) •Time: For <1–18.1 years, average follow-up of 12.4 years	Third National Health and Nutrition Examination Survey (NHANES III) 2474 patients Ethnicity: EA, AA, and Mexican-Americans (Years: 1988–1994, follow-up to 2006)	•Cadmium exposure is a risk factor of cancer mortality in older Americans and the risk increases in those with inadequate zinc intake •Cadmium exposure was not associated with PCa risk	Lin et al.^79^

*EA* European-American, *AA* African-American, *PCA* prostate cancer, *PSA* prostate-specific antigen, *BPH* benign prostatic hyperplasia, *HR* hazard ratio, *OR* odds ratio, *RR* relative risk, *DRE* digital rectal exam, *SNP* single-nucleotide polymorphism, *IGF-I* insulin-like growth factor-I, *IGFBP-3* IGF-binding protein-3, *miRNA* microRNA, *Ctrls* controls

### Silibinin

Over the past two decades, silibinin (flavanolignan from milk thistle “*Silybum marianum*” seeds) has shown strong anti-carcinogenic effects against various types of tumors including PCa.^2,3^ The significant anti-cancer efficacy of silibinin observed in preclinical animal models of PCa led to its transition into a phase II clinical trial to evaluate its bioavailability in patients diagnosed with localized PCa disease.^4^ Prior to surgery, PCa patients either received 13 g of silybin-phytosome for 14–31 days (*n* = 6) or served as untreated control subjects (*n* = 6). High dose of oral silybin-phytosome achieved high plasma concentrations in patients; however, very small amount of silibinin was observed in prostatic tissue due to its short half-life.^4^ Although serum prostate-specific antigen (PSA) levels of patients did not achieve a partial or complete response to silibinin treatment, disease stability was maintained in several patients. Thus, larger clinical studies are still needed to be performed with a more bioavailable form of silibinin to validate its biological efficacy as an effective nutraceutical agent for the clinical management of localized or advanced forms of PCa.

### Grape seed extract (GSE)

GSE is a complex mixture of polyphenols containing procyanidins and their gallate derivatives together known as the proanthocyanidins.^5^ It has shown anti-cancer efficacy against PCa growth and progression in several preclinical models^6^; however, clinical studies have not fully evaluated GSE efficacy in PCa patients. Interestingly, in a 2011 prospective study “VITamins And Lifestyle (VITAL)”^7^ (*n* = 1602; PCa cases; *n* = 35,239 total participants), analyzing the biological outcomes of intake of several dietary supplements (for approximately 5–10 years), GSE consumption stood out as the one associated with reduced risk for PCa (41% reduction in the risk of mortality among PCa patients relative to non-users).^7^ Importantly, we along with a team of medical oncologists have recently initiated a phase II study of GSE product in asymptomatic or minimally symptomatic non-metastatic PCa patients with rising PSA (NCT03087903), wherein GSE efficacy will be examined in a cohort of PCa survivors who have undergone treatment but show signs of rising PSA after local therapies. Given that current clinical studies have not yet identified molecular signatures modulated by GSE in PCa patients and this agent is a widely consumed as a supplement and food additive, more studies are needed to identify synergistic and/or additive interactions of GSE and its constituents with clinically used anti-PCa agents.

### Lycopene and other carotenoids

Lycopene is a powerful carotenoid antioxidant with anti-tumor activity and present in red fruits and vegetables (i.e., tomatoes (tomato-based products), grapefruit, watermelons, and papayas).^8^ Other carotenoids-related compounds such as α-carotene, β-carotene, β-cryptoxanthin, xanthophyll carotenoids, lutein, and retinol have also shown anti-tumor activity. Interestingly, α-carotene, β-carotene, and β-cryptoxanthin can be converted into retinol also known as vitamin A.^9^ Epidemiologic and clinical evidence have shown an inverse relationship between dietary lycopene (including other carotenoids), PCa development and disease progression risk.^9,10^ In a randomized-controlled study, a significant decrease in median PSA levels among PCa patients in the lycopene intervention group relative to control subjects was observed.^11^ Based on non-metastatic PCa patients in the Cancer Prevention Study II Nutrition Cohort, prediagnosis and postdiagnosis dietary lycopene intake did not modify PCa-specific mortality;^12^ however, lycopene intake higher than the median value was significantly associated with 59% lower hazard ratio among high-risk PCa patients. Notably, in a recent prospective study in patients from the Health Professionals Follow-Up cohort, over a 23-year follow-up, average tomato sauce intake was associated with a 46% reduction in risk of *TMPRSS2-ERG*-fusion positive PCa disease.^13^ High plasma levels of cis-lutein/zeaxanthin, all-trans-lycopene, and β-cryptoxanthin after 3 months corresponded to lower PSA levels at 3 and/or 6 months, respectively.^14^ High circulating levels of α-carotene,^10,15^ β-carotene, and total carotenoids lowered PCa susceptibility;^10^ however, circulating levels of lycopene were associated with a non-significant reduction in PCa risk.

Although carotenoids have exhibited preventive effects against PCa, other studies have reported little to no effect and/or antagonistic effect of carotenoid-associated nutraceuticals in PCa susceptibility.^9,12,15–26^ In a large epidemiologic study, lycopene intake was not associated to overall PCa risk; however, retinol, a biosynthesis product of several carotenoids, was linked to a 13% increase (*p* = 0.015) in risk.^9^ Similarly, in the SU.VI.MAX study, a double-blind placebo-controlled and randomized trial, β-carotene had no effect on hazard ratios associated with PCa among 5141 men.^18^ In a cross-sectional study, high serum lycopene levels and total PSA > 2.5 ng/mL were associated with a 1.49-fold increase in PCa risk.^15^ Whereas, serum or plasma levels of retinol, β-carotene, β-cryptoxanthin, lutein and/or zeaxanthin, and total carotenoids did not significantly modify risk.^15,19^ Higher risk of PCa mortality was linked to β-carotene intervention in men relative to non-users in the Alpha-Tocopherol, Beta-Carotene Cancer Prevention (ATBC) Study (*n* = 29,133).^26^ In a prospective study, serum levels of α-carotene, β-carotene, lycopene, retinol did not show a relationship with time to disease progression, treatment, PSA kinetics (i.e., PSA, PSA velocity) and adverse histology in patients with localized disease.^20^ Likewise, another prospective study showed no association between lycopene and β-carotene serum levels and PCa risk in Australian men.^21^ In the PCa Prevention Trial (PCPT), dietary intake of carotenoid excluding lycopene had no effect on the risk of total incident symptomatic benign prostatic hyperplasia (BPH) among placebo arm participants after a 7-year follow-up (*n* = 4770).^17^ In the intervention arm of this study, lycopene also showed no significant association with BPH risk. Notably, carotenoid intervention and circulating levels in relation to PCa development and advanced disease have resulted in null findings in other reports.^12,16,22–25,27^

Lycopene supplementation has shown a modest impact on cellular death, cell cycle, growth, and oxidative stress signaling mechanisms.^28,29^ Recent studies included in this review showed no molecular changes related to other carotenoids. In a Phase II clinical trial, 6 months of lycopene intervention marginally reduced nuclear levels of proliferative marker MCM-2 and cell cycle regulator p27 in benign prostate tissue from patients diagnosed with high-grade prostatic intraepithelial neoplasia (PIN).^28^ After a 3-month lycopene or tomato product intervention, analysis of normal prostate tissue from low-risk PCa patients (Gleason sum ≤ 6) showed apoptotic signaling and nuclear factor (erythroid derived-2) 2-mediated oxidative stress response as the top two ranked pathways altered by treatment compared to placebo.^29^

### Soy isoflavones

Soy Isoflavones are members of the polyphenolic flavonoid family.^30^ These compounds are found in red clover, kudzu root, and soybeans, which are commonly used in Asian and African cuisines. Genistein, daizein, aglycones, equol and glycitein are the predominant isoflavones in soybean and soy-derived food products. Clinical studies on the efficacy of soy intervention have shown some benefits against PCa via its influence on insulin and inflammatory signaling. In a 20-week phase II trial in asymptomatic PCa patients, consumption of soy-almond bread and standard soy bread contributed to a significant increase in IGFBP-3 and decrease in pro-inflammatory cytokines blood levels.^31^ Unfortunately, dietary soy intervention increased serum PSA levels and its doubling time after 126 days with a slight decrease in PSA velocity in PCa patients. Similar to the previous report, isoflavones have exhibited some immunomodulatory properties in the plasma of asymptomatic biochemically recurrent PCa patients.^32^ High plasma genistein concentrations (>640.2 nmo/L) were strongly linked to a 69% decrease in risk of developing PCa among Chinese patients.^33^ At the time of diagnosis, median levels of plasma genistein were significantly lower in PCa patients relative to controls. Short-term administration of isoflavone (80 mg) for 6 weeks in patients also showed an inhibitory effect on cell cycle and apoptotic-associated signaling in prostate tumor tissue.^34^ However, isoflavone intervention did not affect serum levels of total testosterone, free testosterone, PSA, and total cholesterol in PCa patients.

Though limited reports have evaluated genomic evidence for the anti-cancer properties of soy isoflavones in PCa clinical trials, soy supplementation has exhibited some effects on inflammatory, apoptotic, and growth signals in PCa.^31,35,36^ In a pilot randomized double-blinded clinical study, soy isoflavones intervention altered the expression of COX-2, a major molecule in prostaglandin synthesis and cyclin kinase inhibitor p21 in PCa patient prostatectomy specimens.^35^ In the same study, genistein treatment also downregulated COX-2 in both LNCaP and PC-3 cell lines and upregulated 15-PGDH in primary PCa cells. In a 6-month clinical trial, soy protein intervention had no effect on proliferative, and apoptotic molecular markers (i.e., EGFR, Bax:Bcl-2, Bax:PCNA ratios) in high-risk and/or with low-grade PCa disease patients, but alcohol-washed soy protein intake reduced tissue levels of PCNA and Bax in patients relative to milk protein treatment.^36^

### Green and black tea extracts

Green and black tea are extracted from the plant *Camellia sinensis*. (-)-Epigallocatechin-3-gallate (EGCG) is the most abundant and well-studied bioactive polyphenolic constituent of green tea with regard to its anti-cancer properties in several malignancies including PCa.^37^ Theaflavin is the major bioactive polyphenol from black tea; however, it has not been well studied compared to EGCG.^37^ Many population studies have examined the efficacy of green tea in PCa chemoprevention.^23,38–41^ In a recent meta-analysis of 13 clinical studies, green tea catechins demonstrated protective effect against PCa risk.^23^ Concurrent consumption of green tea catechins and natural food products in an adjusted indirect comparison relative to six other natural compounds significantly reduced PCa susceptibility in men diagnosed with high-grade PIN. Daily administration of Polyphenon E (an enriched green tea polyphenol extract) containing 800 mg of EGCG prior to a radical prostatectomy significantly reduced serum levels of PSA in men with cancer-positive prostate biopsies.^38^ However, in another study, Polyphenon E intake showed no significant effects on the serum levels of PSA, insulin-like growth factor, proliferation, and angiogenesis in the prostate tissue of PCa patients after 3–6 weeks.^41^ The efficacy of green tea was also evaluated in patients with localized PCa (*n* = 199) and receiving active surveillance or watchful waiting as clinical management treatment. Oral administration of a capsule containing nutraceuticals, pomegranate, green tea, broccoli, and turmeric for 6 months significantly decreased median PSA percentage levels in patients, regardless of clinical management relative to the placebo group.^39^ Unfortunately, this intervention did not alter Gleason grades in patients.

Tea polyphenols have modulated several molecular signatures in PCa patients. Green tea intake reduced nuclear NFκB in radical prostatectomy tissue and PSA levels in PCa patients compared to black tea and control treatments 3–8 weeks prior to surgical therapy.^40^ Growth factor signaling mediators, HGF, VEGF, IGF, and IGF/IGFBP-3, ratio was decreased in men with PCa.^38,41–43^ Although expression of cell proliferative, apoptotic, and angiogenic markers was not changed by green tea intervention after 3–6 weeks prior to surgery, serum levels of PSA, IGF, and DNA oxidative stress in leukocytes were decreased by treatment in patients.^41^ Also, green and black tea reduced the proliferation of LNCaP PCa cells.^42^ Green tea in a combinatorial nutraceutical intervention promoted oncogenic-related miRNAs in PCa including miR-92-3p that targets PTEN and androgen-regulated miR-125-5p.^43^

### Vitamin D

Vitamin D is a fat-soluble nutraceutical found in dairy, flour, and fortified food products.^44^ It has five isoforms, which include ergocalciferol with lumisterol (D1), ergocalciferol (D2), cholecalciferol (D3), 22-dihydroergocalciferol (D4), and sitocalciferol (D5). Its biosynthesis occurs in the skin in response to solar ultraviolet B radiation exposure. In the body, Vitamin D primarily circulates as 25-hydroxyvitamin D [25(OH)D] and is converted by 1α-hydroxylase into its active form, 1,25-dihydroxyvitamin D [1,25(OH)2D]. Calcium and bone homeostasis are closely dependent on Vitamin D bioavailability. Vitamin D deficiency is linked to elevated susceptibility of PCa development and aggressive disease. Both clinical and other population-based studies have comprehensively evaluated the role of dietary vitamin D as a preventive therapy to reduce disease development and complimentary agent to accepted clinical treatments for PCa. Overall, dietary vitamin D levels have an inverse association to PCa development and disease progression.^45,46^ Vitamin D deficiency (<20 ng/mL) alone increases the risk of PCa development and aggressive PCa in certain subpopulations.^47^ In two large nested case-control studies, vitamin D intervention decreased the risk of developing PCa among patients with high Gleason scores.^48^ In a cross-sectional study, dietary Vitamin D intake showed an inverse association with aggressive PCa (699 cases and 958 controls).^46^ Additionally, high serum levels of vitamin D were correlated with a decrease in PCa risk among men.^48^

Strong expression of calcium-sensing receptor was also related to lethal progression in PCa tumors with low vitamin D receptor (VDR) levels, whereas high VDR-expressing tumors were not linked to disease progression.^49^ In the Malmo Diet and Cancer Study, low PCa mortality was linked to 25(OH)D concentrations (>85 nmol/L),^50^ but lower levels of this metabolite (<50 nmol/L) increased PCa-specific mortality risk in another study.^45^ In a Phase II clinical trial, daily combination therapy of cholecalciferol/vitamin D3 (200,000 IU) and G-2535 (genistein, 600 mg) increased serum levels of calcitriol but this was not reflected in prostate tissues.^51^ Although no increase in calcitrol was observed in prostate, this treatment enhanced androgen receptor (AR) expression and pro-apoptotic effects in prostate tumor tissue compared to placebo-control subjects. In naive metastatic castrate-resistant PCa, a VDR agonist, Inecalcitol, used in combination with docetaxel and prednisone effectively reduced PSA levels in 76% of patients.^52^ Furthermore, high expression of inflammatory markers, serum C-reactive protein (CRP), and interleukin IL-8 were inversely related to 25-(OH)D levels in PCa patients.^53^ Vitamin D3 supplementation (4000 IU daily) promoted higher levels of immune- and inflammation-related genes in US men within 2 months prior to a prostatectomy.^54^ In a nested case-control study of Sweden PCa patients (943 cases), high risk was linked to men in the highest quartile of vitamin D (≥103 nmol/L) with a moderate Gleason score (7) or high serum parathyroid hormone (PTH) levels (≥3.74 pmol/L) as well as men in the highest quartiles of both PTH and calcium (≥2.38 mmol/L).^55^ Lower risk of aggressive and non-aggressive disease was significantly linked to men in the 4th quartile for PTH with low serum levels of Vitamin D (<50 nmol/L). Unfortunately, several population studies have depicted null relationships between vitamin D concentration levels and/or supplementation and PCa susceptibility in median-sized cohorts.^17,22,48,56–61^

Vitamin D treatment has shown some anti-inflammatory and hormone-related molecular changes in PCa.^51,53,62,63^ In Taiwanese PCa patients, genetic variants, HFE rs9393682, and TUSC-3 rs1378033 were associated with time to progression in localized disease and low risk of advanced PCa for patients undergoing androgen deprivation therapy.^62^ Furthermore, *in vitro* studies revealed 1,25-Vitamin D downregulated HFE and when silenced HFE impedes cell proliferation and wound healing. Low expression of TUSC-3 was shown to correspond with poor PCa prognosis in patients, and TUSC-3 silencing enhanced cell migration and growth.^62^ However, 1,25-Vitamin D strongly induced the expression of TUSC-3 in PCa cells. In a Phase II clinical trial, PCa patients with the highest serum levels of prostatic 1,25(OH)2D also had low COX-2 levels, but exhibited high IL-6 levels in their stroma tissue.^63^ Interestingly, 1,25 (OH)2D treatment suppressed TNF-α, IL-6, and IL-8 levels in primary epithelial cells, but only TNF-α and COX-2 levels were downregulated in stromal cells. Similarly, circulating levels of 25(OH)D negatively correlated with pro-inflammatory markers, serum CRP, and IL-8, but NFκB p65-positive cells were elevated in PCa patients.^53^ In early stage PCa, high-dose cholecalciferol and genistein (G-2535) intervention induced AR expression in patient tumor tissue but not benign tissue relative to placebo controls.^51^

### Vitamin E

The protective effect of nutraceutical, Vitamin E, in PCa has been widely studied in published reports and clinical trials.^9^ Tocopherols (α, γ, and δ) possess vitamin E activity and are the most studied vitamin E bioactive constituents in PCa. Vitamin E is fat soluble and found in a variety of foods, such as nuts, seeds, and vegetable oil. Both European and Western diets have a high content of tocopherols. European diets mainly include α-tocopherol, whereas γ-tocopherol is generally present in the Western diets. In a pooled study including 15 cohorts (11,239 cases, 18,541 controls), α-tocopherol consumption was associated with a decrease in risk of PCa overall and aggressive disease susceptibility.^9^ In a small clinical trial, castrate-resistant PCa patients taking APC-100, an antioxidant moiety of α-tocopherol, maintained stable disease and median progression-free survival of 2.8 months.^64^ In a 6-month clinical trial, high α-tocopherol levels were inversely related to serum PSA levels in biochemical recurrent PCa patients (*n* = 39).^14^ In contrast, other studies have shown negative effects associated with vitamin E intervention or its circulating plasma levels in PCa patients. PCa risk was slightly increased in the National Institutes of Health (NIH)-AARP Diet and Health Study due to a high frequency (>7 times per week) of dietary Vitamin E.^65^ High PCa risk estimates were associated with patients who used a dosage of 800 IU per day regardless of frequency. Furthermore, patients with family history of PCa and taking frequent multi-vitamin supplementation (>7 times per week) had a 2.48 and 16.41-fold increase in susceptibility of advanced and fatal PCa disease, respectively. In another report, dietary intake of both α-tocopherols and δ-tocopherols reduced risk of PCa in European-American patients.^66^ Furthermore, positive plasma α-tocopherol levels were also linked to high-grade PCa disease (Gleason grade 7–10).^67^ However, vitamin E intervention combined with low selenium status increased PCa susceptibility in men.^68^ Although vitamin E supplementation has some negative effects in PCa, some large population studies have observed null findings in relation to BPH and PCa.^17,22,66,68–70^

Vitamin E intervention, supplementation, and associated genetic variants have been shown to modulate PCa susceptibility among men.^71–74^ In the multi-center Prostate, Lung, Colorectal, and Ovarian Cancer Screening Trial, rs964184 variant GG genotype located near genes, BUD13, ZNF259, and APOA5, which plays a role in Vitamin E metabolism, showed a protective role against PCa risk with the inheritance of two or more minor alleles.^71^ However, other clinical trials have identified variants such as NKX3.1 rs11781886 linked high risk of developing advanced PCa disease in the presence of Vitamin E intervention.^72^ In the ATBC study cohort, serum levels of tricarboxylic acid cycle, long-chain fatty acid, and glycerophospholipid metabolites were strongly associated with low risk of aggressive PCa with the exception of metabolites, thyroxine, and trimethylamine oxide.^73^ However, these metabolites had no significant interaction with α-tocopherol supplementation. Vitamin E-related transcripts involved in the transport of vitamin E influence PCa susceptibility as well. In a clinical study by Bauer and colleagues (2013), circulating levels of α-tocopherol or γ-tocopherol were associated with disease recurrence; however, superoxide dismutase enzyme 3 (SOD3) rs699473 variant were linked to high-grade PCa, but SOD1 (rs17884057, rs9967983) and SOD2 (rs4880) variants were protective against disease recurrence among men (*n* = 573).^74^ Additionally, SOD1 rs17884057 variant and circulating α-tocopherol levels had a significant interaction with high-grade PCa, but did not remain significant in the highest quartile of α-tocopherol.

### Zinc

Normal prostatic tissue exhibits the highest levels of the mineral zinc compared to any soft tissue in the body.^75^ In earlier epidemiologic studies, dietary zinc demonstrated a protective effect in individuals diagnosed with advanced PCa susceptibility. However, recent reports oppose the preventive role of dietary zinc and contribute to mix findings on the relationship of zinc consumption and PCa risk. Long-term dietary zinc from multi-vitamins or other supplements in PCa patients showed a non-significant 2.0-fold elevation in PCa risk in the US hospital-based Case-Control Surveillance Study (1706 cases, 2404 matched controls).^16^ Epidemiologic evidence has also revealed an interaction between dietary zinc intake and cadmium exposure in relation to PSA levels. In the 2001–2002 National Health and Nutrition Examination Survey, dietary zinc intake less than the median level (<12.67 mg/day) was linked to an increase in creatinine cadmium exposure (1 µg/g).^76^ Cadmium is a non-essential heavy metal that is one of the naturally occurring composites in zinc. This relationship induced a 35% increase in PSA levels in patients. However, this association between cadmium exposure and PSA levels disappears at higher levels of dietary zinc intake.^76^ During the 7-year follow-up period in the PCPT study,^17^ dietary and total zinc intake decreased the susceptibility to develop total symptomatic BPH incidence among the placebo population in the second, fourth, and fifth quintiles.^17^ In the VITAL study, Gonzalez and associates showed that dietary zinc intake over a 10-year period had a non-significant decrease in overall PCa and advanced disease (regional/distant) mortality risk.^77^

Other studies have shown either null and/or possible harmful effects of dietary zinc intake and PCa and/or other associated conditions.^16,17,22,65,77–79^ Varying levels of dietary zinc have been linked to potential PCa risk. Two studies showed that excessive use of multi-vitamin with zinc supplements was associated with high risk of PCa.^16,65^ In the NIH-AARP Diet and Health Study, excessive use of multi-vitamin including zinc was associated with a 4.36-fold increase in the risk of developing fatal PCa among 10,241 men (8765 localized and 1476 advanced cases).^65^ In other studies including the Case-Control Surveillance Study and SU.VI.MAX study, non-significant associations were shown between zinc intake and/or supplementation and circulating levels with PCa susceptibility among European-American, African-American, and Mexican-American men.^16,22,79,80^

Unfortunately, current clinical studies have not evaluated the molecular effects of zinc intervention in PCa. However, two studies have evaluated some aspects of zinc homeostasis affected in PCa. It is well known that zinc levels as well as the expression of zinc transporters are low in PCa and this may impair zinc absorption in the body.^81^ Zinc transporters, hZIP1 and hZIP2, were underexpressed in malignant prostate tissue compared to surrounding normal tissue.^81^ In another study, miR-182 expression in prostate tissue was higher in all PCa cases, but miRs-182 and miRs-346 expression were inversely related to hZIP1 levels in European-American men only.^82^ Overexpression of miRs-183, miRs-96, and miRs-182 reduced intracellular zinc levels and uptake in primary prostatic epithelial cells. Despite recent reports that suggest a possible antagonistic role for zinc in PCa, additional studies are still needed to fully evaluate the efficacy of dietary zinc or supplementation in patients due to limited literature on the molecular and clinical impact of this agent on PCa.

### Selenium

Changes in the physiological levels of the mineral selenium may influence biochemical and metabolic processes in PCa.^83^ Significantly low levels of selenium have been detected in malignant prostatic tissue compared to BPH specimens.^84^ Dietary selenium intervention has been linked to null findings for PCa susceptibility.^16,17,22–25,27,85^ In two meta-analysis studies, selenium intake was related to an increase (though not significant) in PCa risk.^23,86^ In a pooled study of prospective cohorts, the daily intake of 37 supplements including selenium (49–90 μg) did not modify PCa risk in British men.^22^ In the Italian cohort for the Procomb trial (ISRCTN78639965), selenium intervention for 2 years did not significantly change PSA levels, PCa susceptibility, or exhibit a strong association with high PCa mortality risk.^27^ In a nested case control study based on the Physicians’ Health Study and Health Professionals Follow-Up Study cohorts, circulating selenium levels did not significantly modify PCa susceptibility.^24^ No association was observed between circulating levels of selenium and PCa susceptibility considering *TMPRSS2-ERG*-fusion positive or negative cancer stratification relative to control subjects (370 cases, 2740 controls). Over the course of 3–5 years, selenium supplementation of 200 or 400 μg did not affect PSA velocity or risk of PCa mortality in high-risk patients (*n* = 699) in a Phase III randomized, double-blinded placebo-controlled, multi-center trial.^85^

Interestingly, meta-analysis of 17 western population-based studies showed serum selenium was inversely related to PCa susceptibility in individual study cohorts.^86^ Arsenic exposure and plasma selenium levels influence PCa susceptibility in patients. In a case-control study on Taiwanese patients (318 cases and 318 controls), low plasma selenium (≤28.06 µg/dL) and high urinary arsenic concentration (>29.28 µg/L) were associated with elevated PCa risk in multi-variate analyses and significantly interacted with PSA levels (≥20 ng/mL).^87^ In a meta-analysis of 15 prospective studies on PCa patients (4527 cases and 6021 controls), high selenium plasma levels were associated with a lower risk of aggressive PCa and selenium nail content was inversely related to PCa risk, but not for blood selenium.^88^ High toenail selenium levels and selenoprotein P, a major selenium transporter, variants were associated with reduced advanced PCa risk.^89^ In a randomized-controlled trial, tomato product intervention consisting of selenium slightly increased PSA levels among patients with intermediate risk of PCa (*n* = 86).^11^ During this intervention, patients with high levels of lycopene, selenium, and fatty acid C20:5 n-3 combined were linked to a large decrease in serum PSA levels. Selenium intervention has also triggered some inconsistent molecular alterations in PCa. Selenium intervention (300 µg) triggered a reversal of epithelial-to-mesenchymal transition (EMT) via upregulation of epithelial markers and lower expression of mesenchymal-related genes in a Dutch population compared to those receiving the yeast placebo.^90^ Selenium-enriched yeast reduced levels of proteins related to anti-apoptosis (clusterin isoform 1), iron transport/homeostasis (transthyretin, haptoglobin), oncogenesis (α-1B-glycoprotein), inflammatory response (complement component 4B proprotein), oxidative stress (keratin 1), NADH metabolic process (isocitrate dehydrogenase) in healthy African-American and European-American male subjects in a randomized, double-blinded, placebo-controlled clinical trial.^91^ However, selenium upregulated levels of α-1 antitrypsin, a protein involved in hypoxia response, angiotensin precursor, negative regulator of cell growth and proliferation, and albumin, a negative regulator of apoptosis. Seven out of the 11 proteins altered by selenium-enriched yeast play a role in cancer; α-1B-glycoprotein, transferrin, haptoglobin, transthyretin, α-1 antitrypsin, and angiotensin precursor.^91^ In a nested case-control study evaluating the effect of selenium and dietary glucosinolate intake, glutathione peroxidase activity exhibited no significant association to benign hyperplasia risk among men (*n* = 325).^92^ Profluss, a mixture of selenium, lycopene, and saw palmetto tree berries, decreased tissue levels of total interstitial mononuclear cells, B lymphocytes, T lymphocytes, and macrophages after 6 and 3 months in patients with PIN/Atypical small acinar proliferation (ASAP) and BPH, respectively.^93^ Six months of selenium administration combined with lycopene and green tea extract in patients with high-grade PIN and/or ASAP resulted in the upregulation of oncogenic microRNAs and decreased levels of miR-494, which plays a suppressive role in PCa.^43^ Inheritance of selenoprotein-associated variants were related to high-grade,^94^ susceptibility, and disease recurrence in PCa patients, but lost significance after adjustment for multiple comparisons.^95,96^

Other studies have demonstrated a non-protective role of selenium in PCa development and progression.^43,65,67,85,96^ High selenium status in men receiving vitamin E supplements was linked to an increase in low-grade and high-grade disease development risk for PCa.^85^ Also, multi-vitamin use (>7 times/week) including dietary selenium was linked to an increase in the susceptibility of PCa.^65^ Interestingly, a large elevation of 5.8-fold was related to PCa risk and dietary selenium use among fatal cases, but the effect was modest. In a double-blinded randomized clinical study, chemopreventive treatment including high-dose selenium in men (*n* = 30) diagnosed with multi-focal HGPIN and/or ASAP had a higher frequency of PCa incidences after 6 months compared to placebo patients (*n* = 30).^43^ In the SELECT trial, a strong relationship between administration of selenomethionine, a type of selenium supplement, and higher plasma levels of α-tocopherol in men potentially promoted higher PCa hazard risk ratios.^67^ Selenomethionine alone and any selenomethionine supplementation was linked to higher PCa mortality risk among men with high Gleason scores (≥7). Moreover, high mortality risk in patients with the highest plasma levels of α-tocopherol and less than 3 year follow-up to diagnosis was associated with selenomethionine alone, α-tocopherol and selenomethionine or any selenomethionine supplementation.

### Nutraceutical efficacy in diverse populations

To date, majority of nutraceutical chemoprevention/intervention studies have been evaluated in populations of European ancestry. Commonly, higher PCa incidence and fatalities rates have been linked to men with African ancestry. Unfortunately, only a small percentage of studies have examined the effect of nutraceutical intervention on PCa development and progression, and disease traits in diverse populations as shown in Tables 1 and 2. Thus, current clinical and epidemiological studies lack evidence on how effective nutraceutical agents are against PCa in diverse populations. Of the limited studies, majority of these reports have focused on lycopene, selenium Vitamin D, and E supplementation or their circulating plasma levels in relation to PCa risk among men of African, Asian, Latino, and Native Hawaiian descent. In a large prospective cohort study (2015), selenium was related to PCa risk, but adjustment for both selenium and lycopene intake was associated with an increase in risk among Native Hawaiian, European, African, Japanese, and Latino-American men on a 1000 kcal daily diet.^25^ Selenium in African-American patients was linked to high risk of localized, advanced disease, and PCa fatality, whereas Latino men were only linked to localized and low-grade disease. Low PCa risk was associated with high legume intake (≥28.2 g) in Latino men as well.^25^ Selenium had no effect on overall PCa risk regardless of ethnicity. However in the Selenium and Vitamin E Cancer Prevention trial, total, low-grade, and high-grade PCa incidences were higher among African-American men.^97^ Furthermore, African-American men with body mass index of 35 had a 2-fold PCa mortality risk.^91^

The effect of carotenoid circulating patterns and intervention on PCa differs slightly in different ethnic groups. As previously mentioned, adjustment for lycopene intake combined with selenium intake in a multi-ethnic cohort was related to PCa risk, but not when taken alone.^25^ Plasma levels of cis-lutein/zeaxanthin, β-cryptoxanthin, and all-trans-lycopene inversely related to biochemical recurrence in a cohort including African-American men, but in a later study dietary lycopene and β-cryptoxanthin were inversely related to aggressive PCa disease among European and African-Americans, respectively.^98^ Moreover, higher levels of α-carotene and lycopene (cis + trans) were observed in European-American men compared to their African-American counterparts. African-Caribbean men had higher circulating serum levels of α-carotenes and β-carotenes and lutein/zeaxanthin, but lower lycopene/retinol levels due to their dietary intake compared to African-Americans.^99^ Moreover, high PSA levels were marginally linked to low retinol serum levels in Caribbean men. Based on the aforementioned studies, β-cryptoxanthin may have a protective role against PCa in African-American men. The data is suggestive that high α-carotene serum levels may contribute to PCa development in European-American and Caribbean men; however, due to limited epidemiological and clinical data more studies are needed.

Vitamin D deficiency in African-American men has been linked to high risk of PCa and aggressive disease.^47^ Moreover, low plasma 25(OH)D levels in African-Americans was significantly related to PCa aggressiveness, while high calcium intake elevated risk.^48^ However, high dietary Vitamin D intake has been shown to reduce aggressive PCa risk in African-Americans, but this effect was not seen in European-Americans.^46^ Levels of vitamin D metabolites, 25(OH)D and 1,25(OH)2D, vary greatly in PCa; serum levels of 25(OH)D are lower, but prostate tissue levels of 1,25(OH)2D are higher in African-Americans compared with European-Americans.^100^ Also, vitamin D binding protein expression negatively correlated to 25(OH)D serum levels in African-Americans. Although, African-American PCa patients have low serum levels of 25(OH)D, high circulating levels of 25(OH)D (34.27–93.20 ng/mL) are linked to an increase in PCa risk among Jamaican men.^48^ Taken together, the previous studies implicate a possible antagonistic effect of 25(OH)D on PCa development in Caribbean men, but protective role in African-Americans, which warrant additional studies in these populations.

Two major zinc transporters, hZIP1 and hZIP2, have been evaluated in malignant prostate tissue from European-American and African-American men.^81^ Very low levels of both transporters were observed in 92.8% of African-American PCa specimens. However, higher levels of zinc transporters were associated with high-grade prostate carcinoma in European-American men compared to African-Americans. In another study, miR-182 expression was higher in PCa tissue overall regardless of race, but miRs-182 and miRs-346 expression were inversely related to hZIP1 levels in European-American men only.^82^ Overexpression of miRs-183, miRs-96, and miRs-182 reduced intracellular zinc levels and uptake in primary prostatic epithelial cells. However, no differences were observed for hZIP1 expression in African-American and European-American men, but this may be attributed to small sample size.

Furthermore, precision medicine suggests that genetic polymorphisms and socio-economic factors influence PCa susceptibility and must be taken into account for patient care and treatment of diverse populations. For example, the susceptibility of PCa patients to vitamin D supplementation has been modified by the inheritance of genetic variations in certain populations.^49,101,102^ In a meta-analysis of VDR genetic associations with PCa from 2006 to 2016, genetic variant VDR *rs731236* was linked to an elevated risk of PCa development in relation to vitamin D supplementation in Asian-American and African-American men.^101^ Based on 27 case-control studies, VDR gene *Fok I* polymorphism was linked to higher PCa risk in men with European ancestry.^49^ Also, the VDR *rs11568820* variant was related to high risk of aggressive PCa in vitamin D-deficient African-Americans.^49^ In contrast, VDR genetic alterations in exon 4 and 8 and vitamin D intervention were associated with a protective effect against PCa risk in American men, whereas exons 5, 7, and 9 positively associated with disease susceptibility.^103^ VDR-related variants, *HFE (rs9393682)* and *TUSC3 (rs1378033)*, were linked to disease progression in patients with localized tumors (post surgery) and advanced PCA (post androgen deprivation) in two independent cohorts, respectively.^62^ Although both circulating vitamin E plasma levels and intervention have been shown to modulate PCa risk, Vitamin E genetic polymorphisms also influence patient predisposition to develop this malignancy. During vitamin E intervention, inheritance of variants in vitamin E-related genes, *SEC14L2*, *SOD1*, and *TTPA*, significantly modified risk of high-grade PCa in patients.^94^ Two TTPA genetic variants (*rs12679996*, *rs4606052*) were linked to elevated mortality risk via inheritance of the CC genotype in high-grade PCa patients. In another study, the NKX3.1 *rs11781886* variant (CC,CC + CT) genotypes combined with vitamin E or selenium treatment increased PCa risk in a cohort including African-Americans.^72^ Polymorphisms located near *BUD13*, *ZNF259*, and *APOA5* genes play a role in vitamin E transport and metabolism and their associated variants modify PCa risk. For example, in a nested case-control study, the *BUD13 rs964184* variant was linked to a decrease in PCa risk among men of European ancestry (483 cases and 542 controls).^48^ High lycopene levels in PCa patients (Gleason score ≤3 + 4) were linked to lower Fraction of the Genome Altered.^10^ Inheritance of *XRCC1 rs25489* variant and high circulating α-carotene levels in the highest quartile population exerted a protective effect against high-grade PCa in men with the *rs25489* GG genotype. High levels of β-carotene in carriers of the *SOD3 rs699473* TC/CC genotype was associated with low risk of high-grade disease.^10^ Selenoprotein-associated polymorphisms were linked to PCa risk among Dana-Farber Cancer Institute patients (*n* = 722) with localized PCa disease.^96^ Dominant genetic models for *TXNRD2 rs1005873* and *SELENBP1 rs10788804* were significantly linked to an increase in risk of aggressive PCa. Specifically, *TXNRD2* (*rs1005873*, *rs3788310*, and *rs9606174*) variants were linked to higher plasma selenium levels in PCa patients.

Socio-economic status among other social and geographical determinants has been also shown to be associated with elevated PCa incidence and/or mortality in several reports.^104–107^ An inverse relationship exists between socio-economic status and PCa incidence and mortality.^104^ Cancer screening and detection frequently occur earlier in European-Americans (with high socio-economic status) compared to African-Americans with the same socio-economic status. Thus, there is a delay between PCa diagnosis and treatment initiative in African-Americans relative to European-Americans. Moreover, Surveillance, Epidemiology, and End Results (SEER) data showed African-American men had a lower survival rate for surgery and radiation as well as higher incidence of metastasis. Between 2007 and 2011, the SEER registries and US census data showed health insurance had a large effect on disease outcomes for the four leading malignancies in the US.^105^ The most disadvantaged population (median household income $42,885) were linked to a 1.6-fold increase of distant disease and less likely to receive surgical treatment among patients diagnosed with breast, lung, and PCa relative to the high-income population (*p* < 0.001). These differences were observed across quintiles regardless of the insurance status.^105^ Cancer-specific survival was lowest among most disadvantaged patients. Socio-economic status in the VITAL cohort showed a weak association with PCa risk among men.^106^ However, the susceptibility of cancer-specific mortality was significantly increased among men in age-adjusted, sex-adjusted, and demographic-adjusted risk models. African-Americans residing in the Mississippi Delta suffer from higher PCa diagnoses and fatalities compared to average US incidence and mortality rates.^107^ This trend was observed in both rural and urban areas commonly composed of disadvantaged communities. Catchment areas, the surrounding geographic areas and populations that cancer centers service, most often include disadvantaged communities. PCa screening, diagnosis, and treatment access at major cancer centers and hospitals have been shown to differ among certain demographics. The populations serviced within or outside of catchment areas of these cancer centers are associated with lower numbers of African-Americans, Hispanics, and uninsured patients.^108,109^ These patterns may attribute to disadvantaged communities being medically underserved as a result of low socio-economic status or restricted accessibility to medical centers; however, additional studies are needed to validate these relationships.

## Conclusion and future directions

Recent clinical and population-based efficacy studies have shown the potential of nutraceuticals as potent anti-cancer agents; however, genetic alterations, social determinants, population/ethnic, and dosage variations modify the protective effect of these agents. Current clinical and epidemiological evidence in diverse populations has not provided sufficient data to determine whether nutraceutical intervention alone or as complement agents can address therapeutic issues associated with health disparities in PCa. Some nutraceutical agents (i.e., Vitamin D, Vitamin E, zinc, selenium) have been linked to unintended outcomes in relation to PCa susceptibility due to deficiency, and excessive use of supplements. Therefore, caution should be taken for the therapeutic application of these agents. Furthermore, some epidemiologic evidence implicate differences in PCa susceptibility in diverse populations of men based on genetic variants, socio-economic, and environmental factors. Specifically, deficient or low levels of Vitamin D and zinc may contribute to health disparities for African-Americans; however, this is still not well understood in the literature. As neo-adjuvant therapies, several nutraceutical have showed limited, but promising inhibitory effects on pathological features associated with precursor neoplastic lesions in PCa.

Most nutraceutical agents are common constituents in produces or manufactured food products consumed daily by humans and have primarily exhibited non-toxic side effects. Ideally, these agents would serve as potential complement treatment options to overcome toxic side effects exerted by existing clinical therapies against aggressive and advanced PCa in diverse patient populations. Although current clinical and epidemiological evidence identified in this review does not overwhelmingly support the hypothesis of an efficacious and therapeutic role for the selected agents in humans, large efficacy studies for these agents were quite limited and lacked disproportionate populations affected by PCa to demonstrate therapeutic activity in high-risk patients. To fully assess the efficacy of these agents, comprehensive pharmacogenomic, pharmacokinetic, case-control population, and genetic studies and clinical trials are needed to determine low-response and high-response rates to and therapeutic limitations of nutraceuticals among diverse patients. Large-scale and inclusive clinical studies will aid to demonstrate whether agents are best utilized as neo-adjuvant or adjuvant therapies to address genetic and pharmacogenomic vulnerabilities in different populations and overcome health disparities. Moreover, these studies will direct the implementation and design of nutraceutical-driven precision medicine strategies to develop patient-focus therapies, reduce the burden of chemotherapy-associated toxicities, suppress disease resistance, and treat both localized and advanced-stage PCa.
